# Mass testing to end the COVID-19 public health threat

**DOI:** 10.1016/j.lanepe.2022.100574

**Published:** 2023-01-06

**Authors:** Cecile Philippe, Yaneer Bar-Yam, Stephane Bilodeau, Carlos Gershenson, Sunil K. Raina, Shu-Ti Chiou, Gunhild A. Nyborg, Matthias F. Schneider

**Affiliations:** a Institut économique Molinari, France; b Physics Department, Tech Univ of Dortmund, Germany; c New England Complex Systems Institute, USA; d Smart Phases, USA; e Universidad Nacional Autónoma de México, Mexico; f Dr. RP Govt. Medical College, India; g Health and Sustainable Development Foundation, Yilan, Taiwan; h College of Medicine, National Yang Ming Chiao Tung University, Taipei, Taiwan; i World Health Network, International

After a period where many countries have let the SARS-CoV-2 virus spread more or less freely, individuals and communities are now grappling with the many negative health effects and economic ramifications from high levels of illness over long periods. As evidence of the detrimental long-term effects of the virus mount, it is increasingly clear that the policy vacuum comes at an unacceptable price both in the short and long term; its only justification would be if there was no other alternative that did not come at an even greater cost. Entering the cold season, the number of infections will most likely increase significantly in Europe (≈ one – two order of magnitude in 2021). While the world awaits and hopes for new and more effective vaccines, we need tools in the toolbox that can effectively control transmission of rapidly spreading new variants, especially if more pathogenic. Otherwise, we may face significant disruptions and enormous costs due to repeated waves of illness, with each wave increasing the numbers of workers thrown out of the workforce from long term health effects. Lockdowns, due to their social restrictions and high short-term economic costs, are no longer the best available option. We here point out that mass testing (regular asymptomatic screening of the general population) is an alternative approach that can dramatically reduce cases and quickly restore economic and social activity.

## Robustness

A prerequisite to develop a clever strategy to oppose the spread of an infectious disease is knowing where the virus is. In particular, for an asymptomatic infection, testing has been clearly identified as a crucial and potent tool to reduce transmission.^1^, ^2^, ^3^, ^4^ If the virus would be detectable in 100% of the cases as soon as someone is infected, elimination would be simply realized if everyone would just take a test today and quarantine if positive. Although idealized this illustrates the potency and robustness of mass testing already without much mathematical intuition. Reality, however, is not that far off. Mass testing can detect individuals who are just becoming infectious, whether symptomatic, presymptomatic, or asymptomatic. If everyone takes a rapid PCR or LAMP test (nearly as accurate as standard PCR tests and delivers results within 30 min of testing) once per day and those that test positive are then quickly isolated, a 30-fold reduction in the effective reproductive number *R* is possible (for more details, limitations and robustness of our approach see appendix in particular Fig. S2). While some people will test positive before they are symptomatic and others afterward, this large reduction in *R* is possible because, in general, infectiousness is preceded by testability for PCR tests due to the low viral load that can be detected. In contrast, sensitivity for antigen lateral flow tests (LFTs) is lower and individuals may be infectious while still testing negative. However, given their easy accessibility, LFTs can remain a useful tool of surveillance, for instance as a supplement for mass testing, where more accurate tests (PCR or LAMP) are unavailable. If we assume *R* = 10 without testing (or significant other measures), we might expect daily testing to reduce the number of new cases by greater than a factor of 9 each week (see appendix); mass testing would not only reduce *R* but also the serial and generation intervals, which would result in cases decreasing by greater than a factor of 10,000 within one month. This is a tremendous reduction and can be as, or even more effective, than a strict lockdown, which early on was shown to reduce R by a factor of ≈ 5–10. Robustness of elimination due to mass testing of an asymptomatic population is possible even where there is significant non-compliance (appendix II), and conservatively allowing for stochastic false negative rates (appendix II). Elimination can always be recovered by simply increasing the test frequency (1/T) and/or by supporting virus spreading using high-quality masks, implementing HEPA purifiers in schools, restaurants and workplaces or additional, non-invasive surveillance strategies (wastewater, surface or air monitoring).

The numbers above assume everyone is tested once per day. Still, a substantial reduction in cases, although not as dramatic, is possible if testing is slightly less frequent. However, for a test interval of 2–3 days instead of one, the strategy needs to be supplemented or combined with further measures for instance with non-invasive surveillance tests, masking, good ventilation with HEPA filters or social distancing, in particular at high-risk events (see appendix I). On the other hand, as new variants continue to increase the transmission rate, test frequencies higher than daily may be necessary (see Fig. S2). Fortunately, it has been found that gargle-spit sampling is effective and allows taking samples at home, where it can be integrated into daily routines such as tooth brushing in the morning and evening. Also sample collection or drop off can be part of daily procedures for instance by collecting samples at work^5^ or at larger supermarket chains as done in Austria. In order to implement the strategy of mass testing, almost everyone within a certain geographic area will need to be tested regularly. Once that geographic area is cleared of the virus, test frequency can be reduced, further reducing financial costs, time, and efforts spent on testing. Subsequently, testing can be focused on areas that still have community transmission or in which there are new outbreaks.

A benefit of mass testing is that it detects many hidden cases simultaneously and thus shortens the duration of suffering from pandemic interference. Mass testing can be a more successful strategy than contact tracing, especially for airborne transmission. Contact tracing quarantines known contacts, but airborne transmission can lead to individuals who are not known contacts being infected. Where there isn't strong ventilation, airborne particles stay in the air for hours, with transmission occurring through airflow between rooms, including through ventilation systems, as well as presumably even from the aerosolization of viral particles from toilet flushing in shared or public bathrooms.

Elimination can be preserved without strong border controls using general testing twice per week (to achieve R/10 rather than R/30) and daily testing of cross border travelers for two weeks. Non-intrusive HEPA purification in restaurants, retail and workplaces and masking where needed, should continue for robustness in preventing new outbreaks.

## Economy

Mass testing impacts the economy far less than a lockdown, and results in far less social disruption. Calculations by Institut économique Molinari based on OECD quarterly accounts for instance show, that if France, Germany, the EU, or the US were to implement mass testing for every individual every day for a month at 10 dollars a test, the cost for one month of testing would respectively be 12 times, 8 times, 9 and 7 times lower than the loss of GDP per inhabitant measured by comparing the loss of GDP over a year (between 2020 and 2019). Test costs on the order of 10 dollars or even less ^11^ per person can and are being realized using pooling strategies, simple test collection, or new testing methods such as LAMP tests. LAMP tests start at 5 euros per test and pooling five individuals per test can reduce the costs by an additional factor of 5.

This strategy will not only reduce the amount of illness and suffering in the population but likely also still be financially profitable; although society has opened up, the accelerating costs of long COVID, and the increase in prevalence of many other medical conditions after COVID-19, continue to mount with the continuous increase in new cases. The cost of long COVID with today's knowledge was recently estimated to equal the cost of the great recession.^6^

## Feasibility

The feasibility has not only been described repeatedly on theoretical grounds,^1^^,^^3^ but has also been demonstrated practically in several countries and on various scales.^5^^,^^7^ One of the most impressive recent demonstrations for the feasibility of mass testing, however, has probably been rolled out in Germany. In the state of North Rhine-Westphalia, with a population of nearly 18 Million the most populated state of Germany, a pooled PCR test strategy was implemented in 3700 schools and 698 daycare facilities. More than 800,000 individuals were screened twice per week using pooled-RT-PCR, with a total number of swabs of almost 17,000,000 within 13 weeks.^8^ For easy sample acquisition the Lolly method has been introduced, which was very well received, even within daycare facilities, showing that testing can be incorporated into people's daily routines. Importantly, this study also demonstrates the robustness of mass testing. As the sensitivity was still sufficient at pooling of up to 200 people, the pool size is limited by the number of infections only. Indeed, the optimal pool size *N*_*p*_, the size resulting in the minimal number of tests needed to identify a positive case, increases as the number of infections goes down (*N*_*p*_ ≈ *√1/f*, for small *f,* where *f* is the fraction of the population infected). At 100 infections per Million people, the pool size could therefore be increased to ∼100 people per pool. At such low infection rates, the continuous surveillance of an entire German elementary school would therefore produce the costs equivalent to 3 PCR tests a few times a week and even a soccer game, with 40.000 visitors, could be monitored with just a few 100 PCR tests allowing to keep schools, social gatherings as well as the economy open and safe. But there are more examples demonstrating the feasibility of the concept, such as the efforts of the University of Davis and others.^9^ But also the strategies of the NBA (National Basketball League) to create a bubble or during the Olympics in Tokyo are good role models of how a mass testing strategy could be brought to reality.

Sufficiently high rates of mass testing can replace lockdowns as the main tool to reduce transmission. When combined with other measures it helps to achieve the shortest pandemic intervention that can stop social and health related collateral damage and keep the economy open. It is a less expensive, less interfering but highly effective and complementary risk mitigation tool compared to measures such as stay-at-home orders, while ensuring that most people stay healthy. If implemented on a larger scale, mass testing may propel the world towards a real end to this pandemic without the need of lockdowns.

Here we have discussed the opportunity to stop transmission using alternatives to lockdowns. Given the ongoing level of disease and long COVID accumulating in the population, the consequences for health, social stability, and economy even with vaccination are significant and require a reconsideration of the currently prevailing strategy.^10^

## Contributors

All authors contributed to the concept and writing of the manuscript.

## Declaration of interests

The authors declare no competing interests.
