# Supplementary material for: Mass testing to end the COVID-19 public health threat
Source: Lancet Reg Health Eur. 2023 Jan 6;25:100574. doi: 10.1016/j.lanepe.2022.100574 (PMC9816799; doi:10.1016/j.lanepe.2022.100574)
Supplement: Supplementary material [file mmc1.docx]

### **Appendix A: Calculations**

1. NEW INFECTIONS

Let t_0_ be the point in time at which an individual becomes *testable*, i.e. the point in time at which a person would test positive were they to take the test in question due to increase in viral load. We know that some people are testable before they are symptomatic and others become testable afterward, however in general infectiousness is preceded by testability due to the low viral load that can be detected by PCR testing. Antigen Lateral Flow Tests are not as sensitive so individuals may only become testable after infectiousness begins.

Then define τ = t-t_0_, so that *τ* represents the time elapsed since an individual became testable, with negative values representing time before the individual becomes testable. We account for the rare cases of infectious individuals who never become testable below (individuals who never test positive but also never become infectious can be ignored).

Then let *i*(*τ*) be the average infections by an individual as a function of τ, such that, with no isolation/quarantine, $\int_{a}^{b} i(\tau)d\tau$ is equal to the expected number of people an individual will on average infect between *τ* = *a* and *τ* = *b*, with $R=\int_{-\infty}^{\infty} i(\tau)d\tau$ is being the effective reproduction number of the disease without isolation or quarantine.

Now, assume everyone is tested at a frequency where the interval between tests is *T*. Denote *p_n_ = p_n_(T)* as the fraction of individuals, weighted by the expected number of people they will infect, that will never test positive. For small values of *T* (e.g. testing at 24 or 48 hour intervals for a PCR test), we expect *p_n_* to be quite small.

Denote the fraction of infections that occur before an individual tests positive by


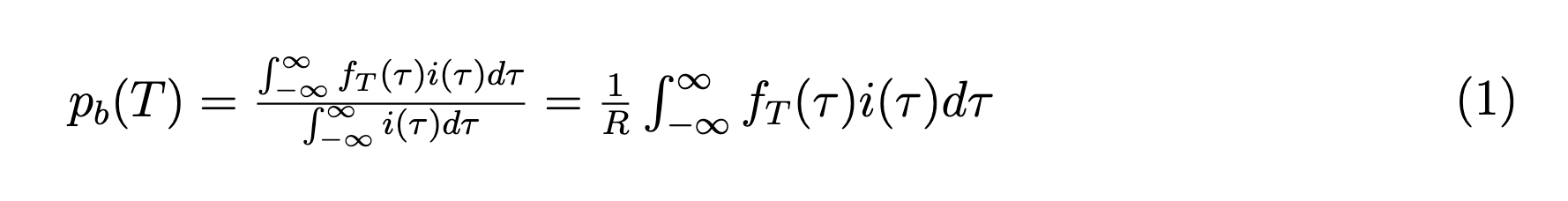


where


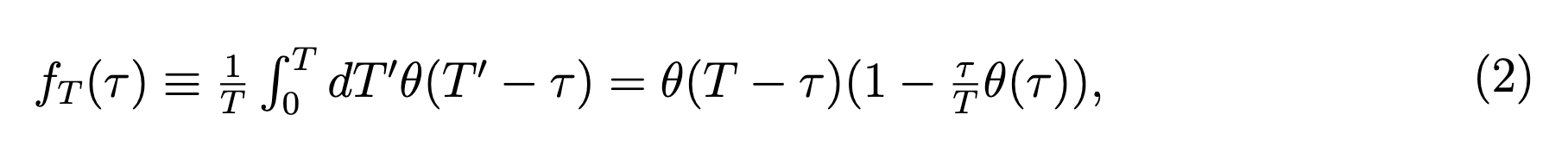


in which *θ* denotes the Heaviside step function. Eq. 1 represents a convolution of the infections i(τ) with the response to these infections f_T_(τ), which is shown in Eq. 2 and Fig. S1.

For small *T*, the dependence of *p_b_* on *T* can be approximated as


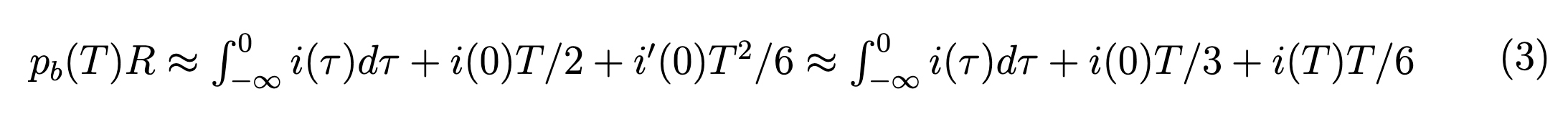


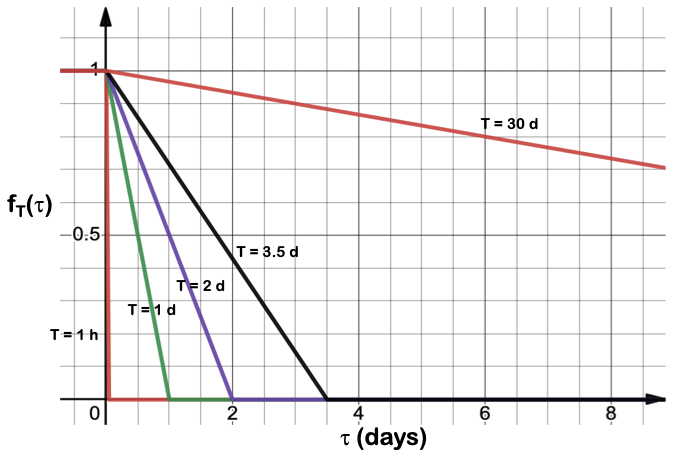


*Fig. S1: The reduction in transmission (response function, f_T_ , see Eq. 2) for different testing intervals (d=days, h=hour). As the interval between tests decreases toward zero or becomes long (see the two extreme cases hourly and monthly (both in red)), f_T_ approaches 0 and 1 fractions of infection respectively. p_b_ (Eq. 1) consequently approaches 0 (practically no infections occur) and 1 (all R infections occur before testing takes place). In the text we discuss the case for 1 and 3 day intervals.*

It would be unusual to be contagious before being testable for PCR tests. If there is any substantial viral RNA in the nose or throat, the PCR test will pick it up. So for PCR tests (though not for antigen tests), *i*(*τ*) ≈ 0 for τ < 0 and so *p_b_*(*T*)*R* is dominated by the i(T)T/6 term.

**Ia. Impact of test frequency on Reff.** The transmissibility *R* divided by the window of infectiousness (how many days over which one is infectious) represents an estimate for the number of cases per day. With a window of infectiousness of roughly 5 days and R ≈ 10 (for Delta and Omicron) we expect *i*(*τ*) ≈ 2 cases per day during the period of peak infectiousness.

1. For a testing interval of *T* = 1 day, if *i*(*T*) = 2, this would lead to *p_b_* = 1/3*R* = 1/30. Multiplying back by *R*, the expected number of expected cases for people testing once per day is 1/3. I.e. the effective reproduction number *Reff*  is significantly smaller than 1, resulting *in rapid exponential decay of cases*. Note that if *i*(*T*) is convex between 0 and *T* as we expect it to be for small *T*, the approximation used is an overestimate as the infectiousness rises rapidly at the end of the period, just before the next testing.
2. For a testing interval of *T* = 3 days, *i*(*T*) = 2 results in an *R_eff_* of *p_b_* = 1/*R* = 1/10, i.e a reproduction of ≈ 1, which - by itself - would not be sufficient to reduce numbers, since there are many sources of imperfection (compliance, test sensitivity etc., see below). This example also demonstrates the robustness of the strategy. Once set in place, increasing the test frequency can counterbalance negative perturbation (perturbations which tend to increase R) and to safely maintain *R_eff_*  < 1.

**Ib. Impact of isolation on** *R_eff_* **.** Assuming an isolation efficiency of *e*, i.e. infectiousness is reduced by a factor of (1 − *e*) once a person tests positive (delays between the time of testing and when isolation begins can be accounted for, see below), then the net effect of the testing and isolation policy will be to reduce the effective reproduction number from *R* to


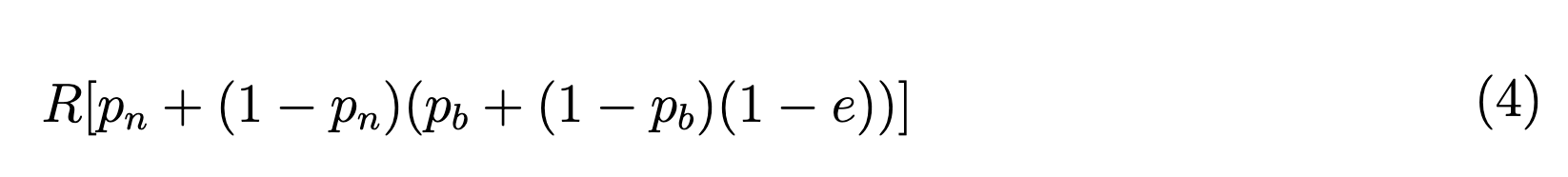


If *p_n_* can be neglected (i.e. almost all infectious individuals eventually test positive) and if *e* is very close to 1, i.e. once isolated, the individual (and any other individuals isolating together with that individual) have very little chance of spreading the virus outside of their bubble (transmission within the bubble will, in this case, have no effect on the effective reproduction number), then the reduced effective reproduction number simplifies to *R_eff_* ≈ *p_b_R* (as indeed assumed above). If one wants to account for a delay of *δ* between when a test is taken and when an individual with a positive result begins isolating, then, in the formula for *p_b_*, f*_T_*(*τ*) should be replaced by f*_T_*(*τ* - *δ*).

**Ic. Impact of compliance on** *R_eff_*  **will be discussed in the next chapter (chapter IIb).**

II. EFFECT OF STOCHASTIC FALSE NEGATIVES AND COMPLIANCE

**IIa False negatives.** A stochastic (random) component of testing may occur even for high viral loads leading to false negative results, due to the process of sampling. For a false negative rate (1 − *f*), the fraction of the infections that happen before testing positive is

*
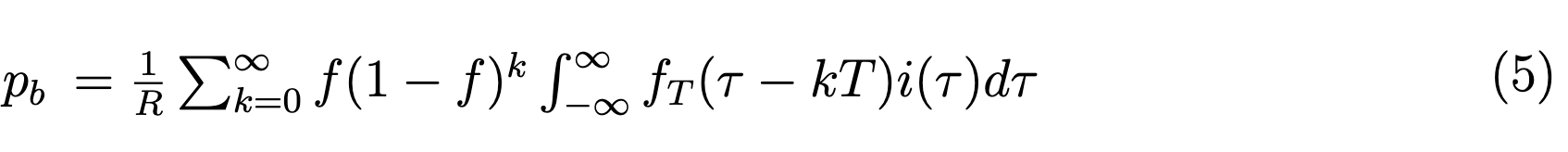
*

For *T* = 1 day, if we assume that *i*(*τ*) ≈ 0 for *τ* < 0 and is bounded above by *i_max_τ*/*T* for *τ* < *T* (and by definition is bounded above by *i_max_* for all *τ*), then we can write:

*
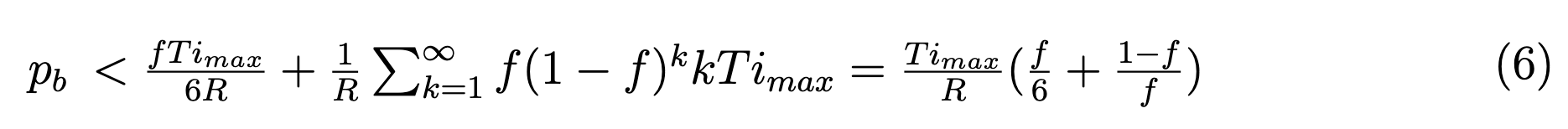
*

The PCR false negative rate at symptom onset is estimated to be 30% for PCR and 50% for antigen tests, however this doesn’t account for the increasing viral load after a person becomes symptomatic. Thus we expect a higher value of *f*. For *f* = 0.9 and 0.8 we would have a factor of 1.56 and 2.3 higher respectively, compared to the previous calculation. For 70%, a conservative lower bound, we have an increase by a factor of 3.3 .

**IIb Compliance.** For a homogeneously mixed population the same treatment (eq. 6, it should be noted, that eq. 6 is an overestimate) can be used to estimate the impact of compliance. A relation between test frequency *T* and compliance *c* (the fraction of the population complying with the test frequency) can be derived for *Reff*  ≈ 1


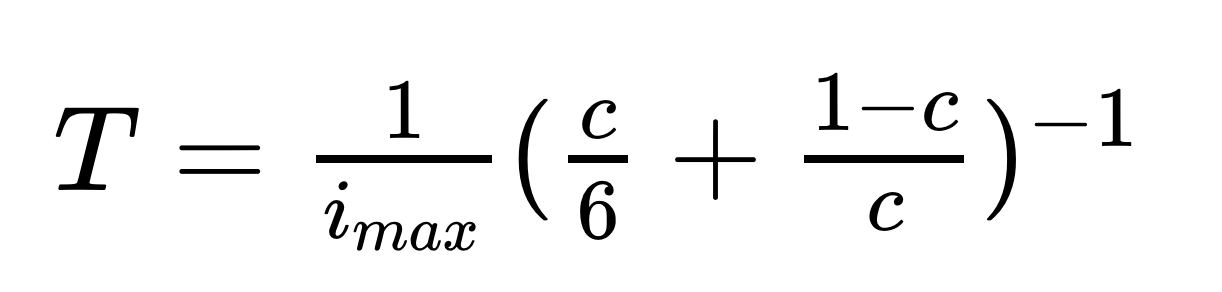


, (6b)

where i_max_ can be estimated to be ≈ 2 here (see *Ia*). In order to reduce *R_eff_* one can therefore either increase compliance or test frequency. The same relation holds also for *T* and the false negative rate *f*, which effectively means the approach does NOT depend on compliance or test quality and is therefore robust against negative external perturbations (**Fig. S2**). If *c* decreases, 1/*T* can be increased, which is feasible once a simple and easy to access test strategy is put in place. If, for instance, compliance drops from 85% to 75% an increase in test frequency from ≈ 4-5x to 6-7x per week is required (for i_max_ ≈ 2).


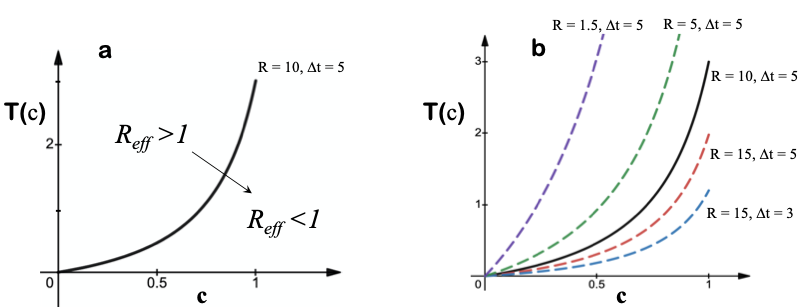


*Fig. S2: Relationship between test frequency* ***T*** *and compliance* ***c****. (****a****) The curve divides the parameter space (T,c) into two (stable and unstable) sections. It illustrates (for imax ≈ 2) how a change in c can be counterbalanced by an adjusted test frequency. (****b****) The stability line shifts towards higher test frequencies (and visa versa) if imax increases either by an increase in R or a decrease in the period of infectiousness Dt. In principle, the same curves would describe the relationship between test frequency and fraction of false negative tests.*

Fig. S2b demonstrates how the test interval required to ensure a robust decrease in infections (*R_eff_* <1) changes with *R* (the reproductive number in the absence of a testing strategy). If no measures are in place *R* equals *R_0_*, the basic reproductive number. While mutations can lead to increase in *R_0_*, measures , such as filtration and ventilation, masks, environmental testing or social distancing ^10^, will support the decrease of *R* (*R*<*R_0_*).

Due to a distribution of incubation periods, individual cases become testable after a period of time that varies from case to case. The distribution of incubation periods is fat tailed with extended incubation periods possible. The expression for the residual number of cases is often estimated by a log normal distribution


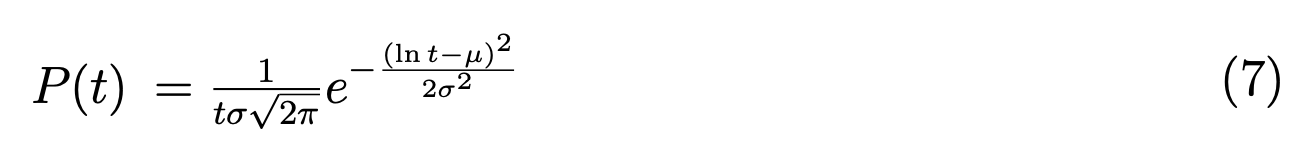


Residual cases are given by


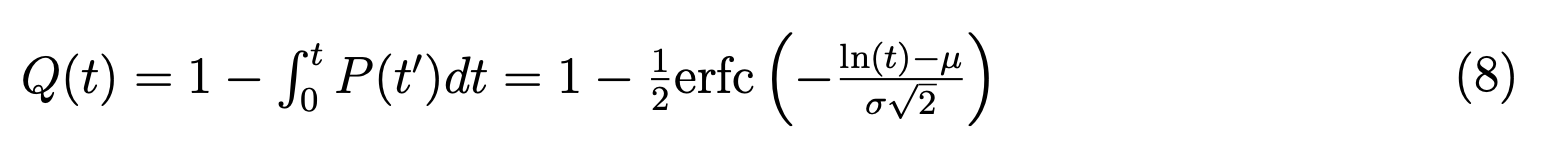


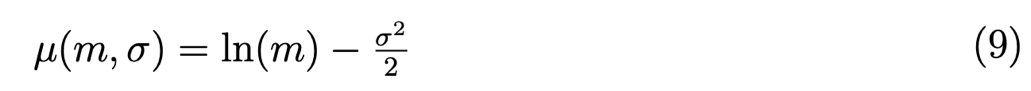


where μ is related to the measured mean of incubation times (m) and sigma (σ) by (McAloon et al BMJ 2020).

With the newer variants there is a trend towards smaller means (Wu et al JAMA 2022) and hence smaller *μ*. This reduces the fraction of long incubation times. Plugging in numbers for *μ* and *σ* we find that the fraction of the tail contribution is ≈ 10% for incubation times more than one week. This places a constraint on the rate at which cases can decline in the population, even given measures that prevent all transmission. For our purpose it is sufficient to note that cases that become testable and infectious in each week can decrease by ≈ 90% or 10-fold each week.

*
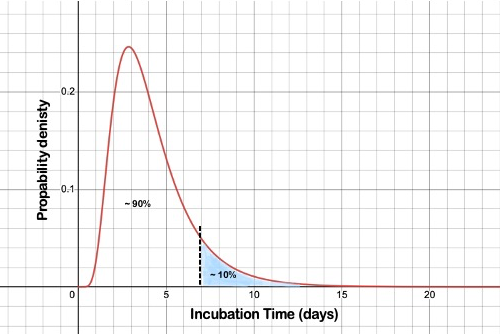
*

*Fig. S3: Distribution of incubation times. The distribution is often described using a log normal, where the long tail accounts for the (sometimes) observed extended incubation times. Detecting all cases up until the blue colored tail part would reduce the number of cases by a factor of 10.*

IV. LIMITATIONS and ROBUSTNESS

**Limitations.** The model and calculations make some basic assumptions in order to enable a transparent analysis and to extract quantitative numbers. We use a mean field approach, which neglects population heterogeneities in composition and time. In applying these results to real world conditions it should be recognized that subgroups of the population will test more frequently than others. Other groups have easier/worse access to tests or are more/less exposed to the virus than others. Further, immunity and virus transmission will also change over time (by waning, mutations or weather changes), leading to heterogeneity in the dynamics. In order to assure robustness of the results, we therefore take a conservative approach, with conservative parameters such as an overestimate of the rate of infection *i(T)* corresponding to the fairly high reference value *R* = *R*_0_ of 10, while the observed population averaged *R* is much lower, suppressed by among other things, immunity (e.g., short term immunity that is limited by waning and variant evolution) and cautious behavior (mandated or voluntarily) of the population. The observed lower population averaged R implies that the testing strategy we propose is robust to our approximations. Finally, by necessity, every testing strategy loses its efficiency the more transmission precedes testability. However, at this time, this is not the case for SARS-CoV-2.

**Robustness**. Key to the concept we present is its robustness. Replacing lockdowns by mass testing not only takes away the tremendous burden of strongly limited interpersonal contact, it also allows adjustment for changes in societal behavior (compliance), transmission (virological changes) as well as virus prevalence (once the virus prevalence is significantly low, test frequency can be reduced). Further, supplementing personal tests with non-invasive test strategies (sewage water, air or surfaces) combined with masks and clean air concepts, can counterbalance such changes leading to a very robust strategy over all with no need for mass lockdowns.

V. ECONOMIC COSTS: TESTING vs LOCKDOWN

The calculation compares the loss of GDP in 2020 relative to 2019 due primarily to lockdowns with the cost of one month of daily testing of the entire population.

The calculation of the loss of GDP per person starts with the fractional decline in real GDP between 2019 and 2020 (Table 1, last column). This amount is multiplied by the 2019 GDP per capita to quantify the loss of GDP per person due to the pandemic in 2020 (Table 2: column A). This loss is divided by the cost of one month of testing per person (30.4 days x US$10 per test, Table 2: column B).

This gives the number of months of daily testing that could have been funded with a budget equivalent to the decline in GDP in 2020 (Table 2: column C). The higher this number, the lower the cost of a month of testing in relation to a year of economic downturn.

**Reference**

McAloon et al (2020). Incubation period of COVID-19: a rapid systematic review and meta-analysis of observational research BMJ Open 10(8).

Wu et al (2022). Incubation Period of COVID-19 Caused by Unique SARS-CoV-2 Strains A Systematic Review and Meta-analysis JAMA Network Open. 5(8)

**Table 1. Economic data (see text (V.) for details)**

A B C

**
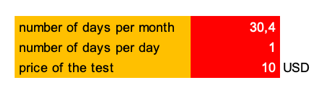
**

**Table 2. Economic data (see text (V.) for details).**

**Appendix B: A More Complete List of References for the Main Text by Topic**

**Robustness: Theory and Experiments**

1.    Peto, J. *et al.* Weekly COVID-19 testing with household quarantine and contact tracing is feasible  and would probably end the epidemic. *R. Soc. open Sci.* **7**, 200915 (2020).

2.    Shen, C. & Bar-Yam, Y. Massive Testing Can Stop the Coronavirus Outbreak. New England Complex Systems Institute. *New Engl. Complex Syst. Inst.* 19–20 (2020).

3.    Kühn, M. J. *et al.* Assessment of effective mitigation and prediction of the spread of SARS-CoV-2 in Germany using demographic information and spatial resolution. *Math. Biosci.* **339**, (2021).

4.    Kühn, M. J. *et al.* Regional opening strategies with commuter testing and containment of new SARS-CoV-2 variants in Germany. *BMC Infect. Dis.* **22**, 333 (2022).

5.    Taipale, J., Kontoyiannis, I. & Linnarsson, S. Population-scale testing can suppress the spread of infectious disease. 1–20 (2021).

6.    Larremore, D. B. *et al.* Test sensitivity is secondary to frequency and turnaround time for COVID-19 screening. *Sci. Adv.* **7**, 1–11 (2021).

7.    Kellner, M. J. *et al.* Head-to-head comparison of direct-input RT-PCR and RT-LAMP against RT-qPCR on extracted RNA for rapid SARS-CoV-2 diagnostics. *medRxiv* 2021.01.19.21250079 (2021).

8.    Poopalasingam, N. *et al.* Determining the reliability of rapid SARS-CoV-2 antigen detection in fully vaccinated individuals. *J. Clin. Virol.* **148**, 105119 (2022).

9.    Ali, S. T. *et al.* Serial interval of SARS-CoV-2 was shortened over time by nonpharmaceutical interventions. *Science (80-. ).* **369**, 1106–1109 (2020).

10.  World Health Network. The 5 pillars of protection. *https://whn.global/guidelines/index.php/Health_Care_Settings* (2022).

11.  Schneider, M. F., Dohmen, L., Hanisch, D. T., Haider, G. & Gruhn, A. The Safe Campus Project&mdash; Resilience of Academic Institutions during the COVID-19 Crisis. *COVID* **2**, 1435–1448 (2022).

12.  Kheiroddin, P. *et al.* How to Implement Safe, Efficient and Cost-Effective SARS-CoV-2 Testing in Urban and Rural Schools within One Month. *COVID* **1**, 717–727 (2021).

13.  ESM. Distribution and collection of PCR Tests Kits in Supermarkets in Austria. *https://www.esmmagazine.com/retail/spar-austria-offers-pcr-test-kits-154408* (2021).

**Aerosol Transmission**

14.  Hwang, S. E., Chang, J. H., Oh, B. & Heo, J. Possible aerosol transmission of COVID-19 associated with an outbreak in an apartment in Seoul, South Korea, 2020. *Int. J. Infect. Dis.* **104**, 73–76 (2021).

15.  Fox-Lewis, A. *et al.* Airborne Transmission of SARS-CoV-2 Delta Variant within Tightly Monitored Isolation Facility, New Zealand (Aotearoa). *Emerg. Infect. Dis.* **28**, 501–509 (2022).

16.  Kang, M. *et al.* Probable Evidence of Fecal Aerosol Transmission of SARS-CoV-2 in a High-Rise  Building. *Ann. Intern. Med.* **173**, 974–980 (2020).

**Economy**

17.  Cutler, D. M. The Economic Costs of Long Covid: An Update. *https://www.hks.harvard.edu/centers/mrcbg/programs/growthpolicy/economic-cost-long-covid-update-david-cutler* 1–4 (2022).

18.  Cutler, D. M. The Costs of Long COVID. *JAMA Heal. Forum* **3**, e221809–e221809 (2022).

**Feasability**

19.  Pavelka, M. *et al.* The impact of population-wide rapid antigen testing on SARS-CoV-2 prevalence in  Slovakia. *Science* **372**, 635–641 (2021).

20.  Dewald, F. *et al.* Effective high-throughput RT-qPCR screening for SARS-CoV-2 infections in children. *Nat. Commun.* **13**, 1–11 (2022).

21.  Pollock, B. H. *et al.* Healthy Davis Together: Creating a Model for Community Control of COVID-19. *Am. J. Public Health* **112**, 1142–1146 (2022).

22.  Matheson, N. J., Warne, B., Weekes, M. P. & Maxwell, P. H. Mass testing of university students for covid-19. *BMJ (Clinical research ed.)* vol. 375 n2388 (2021).

23.  How the NBA conquered COVID-19. *https://andscape.com/features/how-the-nba-conquered-covid-19/* (2020).

24.  Vogels, C. B. F. *et al.* SalivaDirect: A simplified and flexible platform to enhance SARS-CoV-2 testing  capacity. *Med (New York, N.Y.)* **2**, 263-280.e6 (2021).

25.  Akashi, H., Shimada, S., Tamura, T., Chinda, E. & Kokudo, N. SARS-CoV-2 Infections in Close Contacts of Positive Cases in the Olympic and Paralympic Village at the 2021 Tokyo Olympic and Paralympic Games. *JAMA* **327**, 978–980 (2022).

**Consensus Paper**

26.  Lazarus, J. V *et al.* A multinational Delphi consensus to end the COVID-19 public health threat. *Nature* **611**, 332–345 (2022).
